# Assessment of Sleep among Patients with Chronic Liver Disease: Association with Quality of Life

**DOI:** 10.3390/jpm11121387

**Published:** 2021-12-20

**Authors:** Oana-Mihaela Plotogea, Gina Gheorghe, Madalina Stan-Ilie, Gabriel Constantinescu, Nicolae Bacalbasa, Simona Bungau, Camelia Cristina Diaconu

**Affiliations:** 1 Department 5, “Carol Davila” University of Medicine and Pharmacy, 050474 Bucharest, Romania; plotogea.oana@gmail.com (O.-M.P.); gheorghe_gina2000@yahoo.com (G.G.); drmadalina@gmail.com (M.S.-I.); gabrielconstantinescu63@gmail.com (G.C.); 2 Department of Gastroenterology, Clinical Emergency Hospital of Bucharest, 105402 Bucharest, Romania; 3 Department of Visceral Surgery, “Carol Davila” University of Medicine and Pharmacy, Center of Excellence in Translational Medicine, “Fundeni” Clinical Institute, 022328 Bucharest, Romania; nicolae_bacalbasa@yahoo.ro; 4 Department of Pharmacy, Faculty of Medicine and Pharmacy, University of Oradea, 410028 Oradea, Romania; simonabungau@gmail.com; 5 Department of Internal Medicine, Clinical Emergency Hospital of Bucharest, 105402 Bucharest, Romania

**Keywords:** sleep, chronic liver disease, quality of life

## Abstract

The present study aims to assess the sleep characteristics and health-related quality of life (HRQOL) among patients with chronic liver diseases (CLDs), as well as the relationship between them. We conducted a prospective cross-sectional study, over a period of eight months, on patients with CLDs. Sleep was assessed by subjective tools (self-reported validated questionnaires), semi-objective methods (actigraphy), and HRQOL by using the 36-Item Short Form Survey (SF-36) and Chronic Liver Disease Questionnaire (CLDQ). The results indicated that 48.21% of patients with CLDs had a mean Pittsburgh Sleep Quality Index (PSQI) score higher than five, suggestive of poor sleep; 39.29% of patients had a mean Epworth Sleepiness Scale (ESS) score ≥11, indicative of daytime sleepiness. Actigraphy monitoring showed that patients with cirrhosis had significantly more delayed bedtime hours and get-up hours, more awakenings, and more reduced sleep efficacy when compared to pre-cirrhotics. The CLDQ and SF-36 questionnaire scores were significantly lower in cirrhotics compared to pre-cirrhotics within each domain. Moreover, we identified significant correlations between the variables from each questionnaire, referring to HRQOL and sleep parameters. In conclusion, sleep disturbances are commonly encountered among patients with CLDs and are associated with impaired HRQOL. This is the first study in Romania that assesses sleep by actigraphy in a cohort of patients with different stages of CLD.

## 1. Introduction

Quality of life (QOL) represents an important endpoint in healthcare and has been extensively studied in the past decades, especially among patients with chronic diseases [1,2]. Health-related QOL (HRQOL) is a complex concept that was described in various ways, “generally considered to reflect the impact of disease and treatment on disability and daily functioning“ (Mayo’s dictionary, 2016) [3]. 

Sleep health is less frequently defined in the literature compared to HRQOL, and it is mostly expressed in association with its outcomes. There are five main indicators of sleep health, measured either by self-reported and/or objective methods [4,5]:

- Quality (subjectively assessed and divided into “good” or “poor” sleep);
- Duration (time slept over 24 h);
- Efficacy (sleep latency, wake after sleep onset);
- Timing (chronotype—morning vs. evening type);
- Alertness vs. sleepiness.

Based on these indicators, Buysse [5] defined sleep health as “a multidimensional pattern of sleep-wake-fulness, characterized by subjective satisfaction, appropriate timing, adequate duration, high efficiency, and sustained alertness during waking hours“ (Buysse DJ, 2014).

Worldwide, in 2017, chronic liver diseases (CLDs) were estimated to affect 1.5 billion persons, whose diagnoses included non-alcoholic fatty liver disease, viral hepatitis B and C, and alcoholic liver disease [6]. Apart from addressing the morbidity derived from major complications (e.g., liver cirrhosis and cancer), a deep focus has lately been oriented toward sleep disturbances/disorders (SDs) in patients with chronic liver disease (CLD) [7,8,9,10]. It was observed that sleep indicators are impaired in more than half of these patients and that these are independently associated with reduced HRQOL [11].

The current study aimed to assess sleep characteristics and HRQOL among patients with CLDs, starting from the hypothesis that patients with more severe liver disease have poorer sleep indicators and reduced QOL. Second, we intended to examine the relationship between sleep alterations and HRQOL in this population.

## 2. Materials and Methods

### 2.1. Study Design

We conducted a prospective cross-sectional study over a period of 8 months (December 2020−July 2021) in the Clinical Emergency Hospital of Bucharest, Romania, both in ambulatory and hospitalized patients. Convenience sampling was applied as we recruited patients with CLDs who presented for regular follow-ups, or patients who presented for decompensation of their liver disease, taking into consideration the including and excluding criteria which are mentioned below.

### 2.2. Subjects

We included in the study 56 adult patients (older than 18 years) who had been previously diagnosed with a CLD, namely steatosis, hepatitis, or cirrhosis. All patients underwent clinical assessment and laboratory and imaging investigations to establish the diagnosis and differentiate pre-cirrhotic stages from cirrhotic ones. Based on transient elastographic (FibroScan) evaluation, we divided the patients into two subgroups: group 1 (pre-cirrhosis)—including patients diagnosed with CLD who had no/mild/moderate fibrosis (F = 0/F = 1/F = 2−3), and group 2 (cirrhosis)—including patients with cirrhosis and severe fibrosis (F = 4). 

The sociodemographic and clinical variables obtained were the following: gender, age, etiology, and comorbidities (diabetes and cardiovascular disease).

We decided to exclude from the study analysis, due to foreseeable bias/influence, acute hepatitis or acute liver failure, overt hepatic encephalopathy (WEST HAVEN score ≥ 2), known sleep disorders or ongoing treatment with sleep medication, unstable cardiovascular/hemodynamic status (e.g., coma), night-shift workers, patients who did not complete the questionnaires/answer all questions, and patients who did not wear the device 24 h/7 days. There were 9 patients who either answered incompletely (3 patients) or who did not wear the actiwatch at all times for the required period (6 patients), resulting in a dropout rate of 13.84%. Patients who did not complete all the questionnaires argued that the tests were exhaustive, with no personal benefit. Patients who took off the watch either misunderstood the instructions for wearing it or felt uncomfortable with the watch during sleep at night.

### 2.3. Sleep Health Assessment

Sleep was assessed by both subjective (self-reported validated questionnaires) and semi-objective methods (actigraphy) to increase diagnosis specificity and sensitivity. All patients completed the Pittsburgh Sleep Quality Index (PSQI), developed by Buysse et al. from the University of Pittsburgh, using National Institute of Mental Health funding [12]. In addition, the participants completed the Epworth Sleepiness Scale (ESS) [13]. These questionnaires were distributed in the Romanian version by Mapi Research Trust, and were also used before this study in patients with other conditions [14,15]. The PSQI is used to evaluate sleep quality in the previous month and separates “good” sleepers from “poor” sleepers. It comprises 19 items grouped in 7 components: sleep quality, sleep latency, sleep duration, sleep efficacy, sleep disturbances, sleep medication, and daytime dysfunction, each component being evaluated from 0 (no impairment) to 3 (severe impairment). The total score is obtained by summing and it ranges from 0 to 21. Scores higher than 5 points are considered suggestive of “poor” sleepers. ESS identifies patients with daytime sleepiness according to the likelihood of falling asleep in 8 different situations. The scores range from 0 to 24 and, the higher the score, the sleepier the subject. Scores ≥ 11 are considered abnormal [8,16].

Actigraphy is an alternative to polysomnography, being similarly cost-effective but less invasive and easier to use. The actigraph is a wristwatch incorporating an accelerometer that detects subject’s movements [8]. In the present study, the patients were instructed to wear for 7 days an actigraphy wrist device (Actiwatch Philips Respironics; Spectrum Pro, manufactured by Philips Healthcare USA, purchased via LAG MedTech, Kolmar, Sweden). Data were recorded and analyzed by automated Philips Actiware software with standardized reports of bedtime, get-up time, time in bed, total sleep time, onset latency, sleep efficacy, wake time after sleep onset (WASO), and number of awakenings per night. 

### 2.4. HRQOL Assessment

HRQOL was assessed using the 36-Item Short Form Survey (SF-36) and Chronic Liver Disease Questionnaire (CLDQ). SF-36 is a self-related questionnaire which contains 36 multiple-choice questions indicating overall physical and mental health status. The questions are grouped into 8 domains: physical functioning, role limitations because of physical health, role limitations because of emotional problems, body pain, general health, energy/fatigue, social functioning, and emotional well-being. The scores can range from 0 to 100 and the lower the score, the more altered the HRQOL [17]. CLDQ is a self-reported questionnaire which assesses the HRQOL of patients with CLDs. It comprises 29 questions grouped in 6 domains: abdominal symptoms, fatigue, systemic symptoms, emotional function, worry, and activity. CLDQ evaluates the symptoms which occurred over the last two weeks before completion, each domain being scored from 1 to 7. The total score is obtained as the mean value of the six domains and a higher score corresponds to a better QOL [18].

### 2.5. Ethics

The study was approved by the Research Ethics Committee of the Clinical Emergency Hospital of Bucharest, Romania (approval no. 3928/12.04.2021) and all participants provided written, informed consent for wearing the actigraph, while completing the questionnaires was considered implied consent to participate. The study was conducted according to the Declaration of Helsinki (1975), as revised in 2008, for medical research involving human subjects [19]. 

### 2.6. Statistical Analysis

Data were collected in Microsoft Excel and statistically analyzed with the IBM SPSS v.20 software package program. Descriptive analysis was performed for the prevalence of poor sleep and reduced HRQOL in the study groups and comparison of demographic and clinical variables between groups. Continuous variables were expressed as means ± standard deviations and ranges or as medians and ranges. Categorical variables were expressed as frequencies/absolute numbers with percentages. A two-sided *p*-value of <0.05 was indicative of statistical significance.

## 3. Results

### 3.1. Background Patients’ Caracteristics

A total of 56 patients were enrolled during the study period and their baseline characteristics are depicted in Table 1. There were 41 males and 15 females, with a mean age of 59.75 ± 10.06 years. Alcoholic liver disease was the most predominant etiology (37.50%), followed by chronic viral hepatitis (30.40%). There were 11 patients with mixed etiology, both viral and alcoholic, and 7 patients who had been diagnosed with non-alcoholic fatty liver disease (NAFLD). Regarding comorbidities, we interviewed patients whether or not they had diabetes mellitus and/or cardiovascular disease. An important number of patients were known to have cardiovascular disease (19 patients) and diabetes (17 patients). 

We further divided the entire group in two groups:

- Group 1: 23 patients with pre-cirrhosis (patients with steatosis and chronic hepatitis with Fibro Scan results that revealed no/mild/moderate fibrosis (F ≤ 3);
- Group 2: 33 patients with cirrhosis (F = 4).

Patients with cirrhosis had a significantly higher mean age than pre-cirrhotic patients (62.39 ± 8.05 vs. 55.96 ± 11.50 years, *p* < 0.05, ANOVA). There were 11 cases with compensated cirrhosis and 22 with decompensated stages, comprising ascites, jaundice, and upper gastrointestinal bleeding, and none had clinical hepatic encephalopathy, as this represented an exclusion criterion.

### 3.2. Sleep Assessment–Patients’ Characteristics

We evaluated sleep characteristics of the patients included in the study through self-administered questionnaires (PSQI and ESS) and also by a semi-objective method: actigraphy (Table 2). The overall results showed that 48.21% of patients with CLD had a mean PSQI score higher than 5, suggestive of poor sleep. Moreover, 39.29% of all patients included in the study had a mean ESS score ≥ 11, which indicates that daytime sleepiness is also frequent. 

When comparing the PSQI and ESS mean scores between the two groups, we noticed that cirrhotic patients had a significantly higher prevalence of daytime sleepiness evaluated by ESS score (9.73 ± 4.80 vs. 6.30 ± 5.14, *p* = 0.014, ANOVA). Poor sleepers (PSQI score > 5) were more prevalent among cirrhotic patients (54.55%), but without statistical significance compared to pre-cirrhotics (39.13%). 

Actigraphy monitoring showed that patients with cirrhosis had a significantly more delayed bedtime hour and also get-up hour when compared to pre-cirrhotics. Consequently, they also spent more time in bed, even though sleep time was similar between the two groups. Sleep efficacy, evaluated as a mean percentage of the entire group of patients with CLD, was 80.85 ± 4.67%, considered as normal (the cut-off being 80%). However, we observed a statistically significant difference when we compared the results in the two groups: 78.51 ± 3.11% in cirrhotics vs. 84.20 ± 4.55% in pre-cirrhotics (*p* < 0.001, ANOVA). The number of awakenings per night was additionally registered and it revealed a significantly higher mean number among cirrhotic patients compared to pre-cirrhotic patients (40.47 ± 10.01 vs. 28.18 ± 11.88, *p* < 0.001, ANOVA). There was no significant difference between groups regarding the total sleep time, onset latency, or WASO. 

By comparing patients with compensated cirrhosis with those with decompensated cirrhosis, we noticed a significant difference regarding PSQI and ESS scores, with 72.73% of the decompensated cirrhotics having a poor sleep quality and 63.64% of them having severe daytime sleepiness (Table 3). Moreover, actigraphic monitoring demonstrated that decompensated stages were associated with significantly more awakenings during night (44.93 ± 6.80), compared to compensated stages (31.55 ± 9.60), and reduced overall sleep efficacy (77.36 ± 2.29 vs. 80.82 ± 3.33).

### 3.3. Predictors of Poor Sleep and Daytime Sleepiness

In order to determine the predictors of poor sleep and daytime somnolence, we used simple and multiple logistic regression analysis. Age, etiology, diabetes, and cardiovascular disease were included as variables in the multiple regression; however, only age was an independent predictor of a poor sleep (PSQI < 5). Therefore, with every year, a patient with CLD has an 18% chance to be categorized as a poor sleeper (Table 4). 

Furthermore, using the same multivariate analysis of variance as above, we included the variables age, diabetes, and cardiovascular disease to evaluate the predictors of daytime somnolence. Both age and diabetes were independently associated with ESS ≥ 11 (Table 5).

### 3.4. HRQOL Assessment—Patients’ Characteristics

We administered two questionnaires to investigate the HRQOL among patients with CLDs (Table 6). The CLDQ total score was 3.90 ± 1.59 for all patients, with significantly lower scores in cirrhotics compared to pre-cirrhotics, both in total score and within each domain. Abdominal symptoms were the lowest rated items (2.77 ± 1.10) of complaint in patients with cirrhosis, followed by systemic symptoms (3.04 ± 1.03) and worry (3.07 ± 1.28). 

For the SF-36 questionnaire, the subdomain “general health” registered the lowest score, with noticeable differences between the two groups. Cirrhotic patients experienced significantly more body pain and physical functioning limitations because of physical health problems than pre-cirrhotic patients. 

We further investigated the HRQOL, comparing compensated cirrhotics with decompensated stages and observed significantly lower scores in all subdomains completed by patients with a more severe disease (Table 7). From the specific questionnaire (CLDQ), the deepest impact was given by abdominal symptoms, followed by worry, while SF-36 revealed the lowest score in the general health section, followed by limitations because of physical health problems.

### 3.5. Associations between Sleep Characteristics and HRQOL among Enrolled Patients

We identified significant correlations between variables from each questionnaire, both the HRQOL and sleep assessment (Table 8). We included the PSQI and ESS scores as well as two parameters recorded by actigraphy: sleep efficacy and episodes of awakenings/night. Patients with CLDs who expressed low scores on the two questionnaires about HRQOL also experienced high scores for PSQI and ESS, indicative of poor night-time sleep and daytime sleepiness. The sleep efficacy proved to be good in patients with high HRQOL parameters, while a high number of awakenings was associated with a reduced HRQOL. The strongest effect was observed with physical functioning, which was the lowest for patients who experienced the highest PSQI and ESS scores. Moreover, high activity scores—a subdomain of CLDQ—were strongly correlated with good sleep efficacy and reduced episodes of awakenings. 

**Figure 1:**
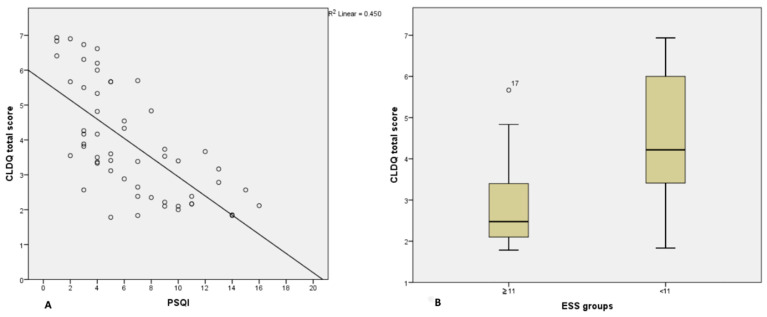
Scatter Plot representing the relationship between PSQI scores and CLDQ scores (**A**) and Box Plot representing the relationship between ESS scores and CLDQ scores (**B**); Abbreviations: PSQI = Pittsburgh Sleep Quality Index, CLDQ = chronic liver disease questionnaire, ESS = Epworth Sleepiness Scale.

**Figure 2:**
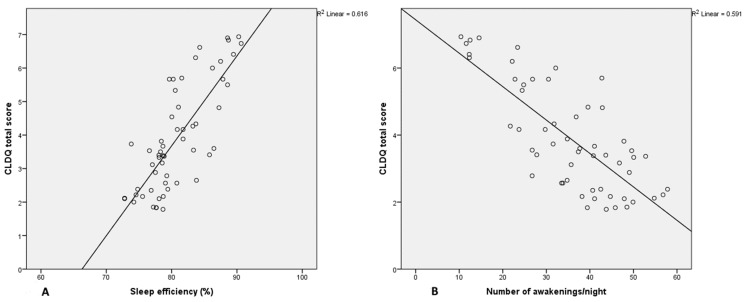
Scatter Plot representing the relationship between sleep efficacy (**A**) and number of awakenings per night (**B**) and CLDQ scores. Abbreviations: CLDQ = chronic liver disease questionnaire.

Figure 1 and Figure 2 show the relationship between total CLDQ score and results obtained by sleep questionnaires and actigraphic monitoring. Most patients with high CLDQ scores had low PSQI and ESS scores (Figure 1A,B), while patients with the lowest sleep efficacy and most frequent awakenings reported the greatest impairment in HRQOL (Figure 2A,B).

## 4. Discussion

This prospective ongoing study is the first to assess sleep disorders among Romanian patients with CLDs by using actigraphy and correlate its results with subjective tools for sleep quality and HRQOL. 

Sleep disorders have been previously described in patients with CLDs in several studies [8,10,11,20,21,22], where their prevalence varies widely from 47% to 81%, mainly due to different assessment methods, heterogenous population, and cumulative influencing/bias factors (e.g., coffee intake, alcohol, sleep medication, presence of hepatic encephalopathy, associated comorbidities, etc.). We reported in our study, among CLD patients, a prevalence of 48.21% of nighttime disturbances and 39.29% of daytime sleepiness, evaluated by PSQI and ESS, respectively. The scores for both questionnaires were significantly higher in decompensated patients, showing a direct relationship between impaired sleep quality and daytime somnolence, and complicated, severe liver disease. Excessive daytime sleepiness has been considered a feature of hepatic encephalopathy, since ESS score has been shown to correlate with the degree of hepatic encephalopathy [16,23,24,25]. Still, we demonstrated that daytime somnolence is present in a high percentage even in pre-cirrhotic patients. This finding may indicate a possible early minimal hepatic encephalopathy (HE) before becoming clinically evident in patients with cirrhosis, however, of course, further prospective studies are needed to confirm this hypothesis.

In addition to the subjective data of sleep quality, we added objective measures of sleep characteristics by using actigraphy. Studies from the literature show that patients with cirrhosis, in particular, experience “delayed sleep phase syndrome” [26], with prolonged onset latency, poor sleep efficacy, and fragmented sleep with frequent awakenings [16,20,26]. This information is also supported by our study, which showed delayed bedtime and get-up hours, lower sleep efficacy, and also more awakenings in patients with cirrhosis compared to pre-cirrhotic ones. Controversially, our study failed to reveal significant differences of onset latency and total sleep time between pre-cirrhotic and cirrhotic patients, as the periods were similar. Moreover, our evidence shows that the difference in sleep parameters is even more important when comparing decompensated stages with compensated forms.

HRQOL in patients with CLDs is influenced by various factors. On one hand, patients experience multiple symptoms related to liver disease, such as itching, fatigue, weight loss, and “fuzzy-thinking”, which can also interfere with their social life. On the other hand, psychological distress strains on patients with advanced stages, when concerns regarding disease progression tremendously impact their QOL [27]. All these factors are also contributors to sleep abnormalities. However, researchers investigated the relationship between sleep impairment in patients with cirrhosis and HRQOL independently of other factors [11,16]. An important finding of our study showed that, besides cirrhotics, patients in pre-cirrhotic stages also experience reduced QOL, directly influenced by poor sleep quality. 

The study has a series of limitations. First of all, it has been conducted in an emergency hospital, where decompensated cirrhosis represented a high percentage of the patients enrolled. Secondly, the study was based on a single assessment and exclusively among patients with a diagnosis of CLD, lacking a normal control group. However, the aim of the study was to investigate sleep and HRQOL in a population with presumed abnormalities. Third, the study did not track either the medication, nor the reasons of decompensation, which might have explained the significant difference between compensated and decompensated patients regarding sleep parameters and HRQOL scores. Further studies are needed to elucidate the contributing factors and their pathogenesis.

Finally, we need to mention the limits given by the subjective and semi-objective methods (questionnaires and actigraphy) that we used to assess sleep and HRQOL. These evaluations, especially questionnaires, are predisposed to bias as they might be overestimated by the patients. Therefore, an objective method is advisable to support the evidence, namely polysomnography, which would definitely offer a valuable extension of our work into future prospective studies.

## 5. Conclusions

Sleep disturbances are commonly encountered among patients with CLDs and are associated with impaired HRQOL. In the present study, we demonstrated that the more severe the liver disease, the poorer that sleep and QOL are. Moreover, this is the first study in Romania that assessed sleep by actigraphy in a cohort of patients with different stages of CLDs. 

## Figures and Tables

**Table 1:** Demographic and clinical data of patients with CLDs.

Demographic and Clinical Data	All Patients (*n* = 56)	Pre-Cirrhosis (*n* =23)	Cirrhosis		
			Total (*n* = 33)	Compensated (*n* = 11)	Decompensated (*n* = 22)
Age (mean ± SD)	59.75 ± 10.06	55.96 ± 11.50 *	62.39 ± 8.05 *	59.27 ± 7.24	63.95 ± 8.13
Gender (males), *n* (%)	41 (73.20%)	16 (69.60%)	25 (75.80%)	8 (72.70%)	17 (77.30%)
Etiology, *n* (%)					
Alcoholic	21 (37.50%)	7 (30.40%)	14 (42.40%)	5 (45.40%)	9 (40.90%)
Viral hepatitis	17 (30.40%)	9 (39.10%)	8 (24.20%)	4 (36.30%)	4 (18.20%)
Alcoholic + Viral Hepatitis	11 (19.60%)	2 (8.70%)	9 (27.30%)	2 (18.20%)	7 (31.80%)
NAFLD	7 (12.50%)	5 (21.70%)	2 (6.10%)	0 (0%)	2 (9.10%)
Diabetes, *n* (%)	17 (30.40%)	6 (26.10%)	11 (33.30%)	2 (18.20%)	9 (40.90%)
Cardiovascular disease, *n* (%)	19 (33.90%)	9 (39.10%)	10 (30.30%)	2 (18.20%)	8 (36.40%)

NAFLD = non-alcoholic fatty liver disease; * *p* < 0.05, ANOVA.

**Table 2:** Sleep assessment among patients with CLDs.

Sleep Parameters	All Patients (*n* = 56)	Pre-Cirrhosis (*n* = 23)	Cirrhosis (*n* = 33)	*p*-Value
PSQI (mean ± SD)	6.50 ± 3.90	5.65 ± 3.57	7.09 ± 4.06	0.177
Good sleepers (≤5), *n* (%) Poor sleepers (>5), *n* (%)	29 (51.79%)	14 (60.87%)	15 (45.45%)	0.194
	27 (48.21%)	9 (39.13%)	18 (54.55%)	
ESS (mean ± SD)	8.32 ± 5.18	6.30 ± 5.14	9.73 ± 4.80	0.014 *
≥11, *n* (%) <11, *n* (%)	22 (39.29%)	7 (30.43%)	15 (45.45%)	0.197
	34 (60.71%)	16 (69.57%)	18 (54.55%)	
Bed time (hour: minutes ± SD)	22:26 ± 0:48	22:09 ± 0:47	22:38 ± 0:45	0.025 *
Get-up time (hour: minutes ± SD)	7:46 ± 0:55	7:04 ± 0:37	8:15 ± 0:45	<0.001 *
Time in bed (hour: minutes ± SD)	9:19 ± 0:51	8:54 ± 0:47	9:36 ± 0:46	0.002 *
Total sleep time (hour: minutes ± SD)	7:36 ± 0:40	7:34 ± 0:40	7:38 ± 0:40	0.752
Onset latency (minutes ± SD)	19.43 ± 8.27	17.91 ± 9.09	20.49 ± 7.61	0.253
Sleep efficacy (% ± SD)	80.85 ± 4.67	84.20 ± 4.55	78.51 ± 3.11	<0.001 *
WASO (minutes ± SD)	38.69 ± 8.22	38.74 ± 9.60	38.65 ± 7.27	0.966
Number of awakenings per night (mean ± SD)	35.42 ± 12.33	28.18 ± 11.88	40.47 ± 10.01	<0.001 *

PSQI = Pittsburgh Sleep Quality Index; ESS = Epworth Sleepiness Scale; WASO = Wake time After Sleep Onset; * *p* < 0.05, ANOVA.

**Table 3:** Comparison between compensated and decompensated cirrhosis regarding sleep assessment.

Sleep Parameters	Compensated (*n* = 11)	Decompensated (*n* = 22)	*p*-Value
PSQI (mean ± SD)	4 ± 2	8.64± 3.97	0.001 *
Good sleepers (≤5), *n* (%)	9 (81.82%)	6 (27.27%)	0.004 **
Poor sleepers (>5), *n* (%)	2 (18.18%)	16 (72.73%)	
ESS (mean ± SD)	6 ± 3	11.59 ± 4.46	0.001 *
≥11, *n* (%)	1 (9.10%)	14 (63.64%)	0.004 **
<11, *n* (%)	10 (90.90%)	8 (36.36%)	
Bedtime (hour: minutes ± SD)	22:35 ± 0:45	22:40 ± 0:45	0.811
Get-up time (hour: minutes ± SD)	8:05 ± 0:39	8:20 ± 0:48	0.391
Time in bed (hour: minutes ± SD)	9:29 ± 0:40	9:40 ± 0:50	0.544
Total sleep time (hour: minutes ± SD)	7:46 ± 0:38	7:34 ± 0:42	0.421
Onset latency (minutes ± SD)	18.34 ± 5.83	21.57 ± 8.26	0.256
Sleep efficacy (%± SD)	80.82 ± 3.33	77.36 ± 2.29	0.001 *
WASO (minutes)	37.48 ± 7.11	39.23 ± 7.44	0.524
Number of awakenings per night (mean ± SD)	31.55 ± 9.60	44.93 ± 6.80	<0.001 *

PSQI = Pittsburgh Sleep Quality Index; ESS = Epworth Sleepiness Scale; WASO =Wake time After Sleep Onset; * *p* < 0.05, ANOVA; ** *p* < 0.05, Pearson Chi-square.

**Table 4:** Logistic regression analysis for predictors of poor sleep (PSQI > 5).

Logistic Regression Analysis for Predictors of Poor Sleep (PSQI > 5)						
	Simple Regression			Multiple Regression		
Variables	Poor Sleepers (*n* = 27)	Good Sleepers (*n* = 29)	*p*-Value	OR [95% CI]	β Coef.	*p*-Value
Age (mean ± SD)	66.59 ± 7.02	53.38 ± 8.13	<0.001 *	0.828 [0.725–0.945]	−0.189	0.003 ***
Gender (males), *n* (%)	19 (70.40%)	22 (75.90%)	0.765	-	-	-
Etiology, *n* (%)			0.027 **			
Alcoholic	10 (37%)	11 (37.90%)		REF		
Viral Hepatitis	4 (14.80%)	13 (44.80%)		2.687 [0.096−75.017]	0.988	0.561
Alcoholic+Viral Hepatitis	7 (25.90%)	4 (13.80%)		3.913 [0.156–98.37]	1.364	0.407
NAFLD	6 (22.20%)	1 (3.40)		3.024 [0.101–90.437]	1.107	0.523
Diabetes, *n* (%)	15 (55.60%)	2 (6.90%)	<0.001 **	4.531 [0.458–42.354]	1.511	0.185
Cardiovascular disease, *n* (%)	16 (59.30%)	3 (10.30%)	<0.001 **	0.930 [0.103–8.419]	−0.073	0.948

NAFLD = non-alcoholic fatty liver disease; * *p* < 0.05, ANOVA; ** *p* < 0.05, Pearson Chi-square; *** *p* < 0.05, ANOVA.

**Table 5:** Logistic regression analysis for predictors of daytime somnolence (ESS ≥ 11).

Logistic Regression Analysis for Predictors of Daytime Somnolence (ESS ≥ 11)						
	Simple			Multiple		
Variables	ESS ≥ 11 (*n* = 22)	ESS < 11 (*n* = 34)	*p*-Value	OR [95% CI]	β Coef.	*p*-Value
Age (mean ± SD)	68.32 ± 5.28	54.21 ± 8.40	<0.001 *	0.776 [0.641–0.940]	−0.254	0.009 ***
Gender (males), *n* (%)	15 (68.20%)	26 (76.50%)	0.351	-	-	-
Etiology, *n* (%)			0.059			
Alcoholic	8 (36.20%)	13 (38.20%)		-	-	-
Viral hepatitis	3 (13.60%)	14 (41.20%)		-	-	-
Alcoholic+Viral Hepatitis	6 (27.30%)	5 (14.70%)		-	-	-
NAFLD	5 (22.70%)	2 (5.90%)		-	-	-
Diabetes, *n* (%)	15 (68.20%)	2 (5.90%)	<0.001 **	13,311 [1.253–141.4]	2.589	0.032 ***
Cardiovascular disease, *n* (%)	16 (59.30%)	3 (10.30%)	<0.001 **	2.525 [0.321–19.86]	0.926	0.379

NAFLD = non-alcoholic fatty liver disease; * *p* < 0.05, ANOVA; ** *p* < 0.05, Pearson Chi-square; *** *p* < 0.05, ANOVA.

**Table 6:** Assessment of QOL among patients with CLDs.

HRQOL Parameters	All Patients (*n* = 56)	Pre-Cirrhosis (*n* = 23)	Cirrhosis (*n* = 33)	*p*-Value
**CLDQ (mean ± SD)**				
Total score	3.90 ± 1.59	4.98 ± 1.64	3.15 ± 1.06	<0.001 *
Abdominal symptoms	3.55 ± 1.66	4.67 ± 1.71	2.77 ± 1.10	<0.001 *
Fatigue	3.86 ± 1.70	4.96 ± 1.81	3.09 ± 1.10	<0.001 *
Systemic symptoms	3.93 ± 1.76	5.21 ± 1.81	3.04 ± 1.03	<0.001 *
Activity	4.03 ± 1.81	5.27 ± 1.71	3.17 ± 1.33	<0.001 *
Emotional function	4.35 ± 1.53	5.18 ± 1.56	3.78 ± 1.24	<0.001 *
Worry	3.70 ± 1.71	4.60 ± 1.86	3.07 ± 1.28	0.001 *
**SF-36 (%, mean ± SD)**				
Physical functioning	74.10 ± 21.76	85.00 ± 21.05	66.51 ± 19.10	0.001 *
Role limitations due to physical health problems	65.71 ± 26.10	75.00 ± 23.83	59.24 ± 25.98	0.025 *
Role limitations due to emotional problems	61.91 ± 23.30	66.67 ± 24.63	58.59 ± 22.11	0.205
Energy fatigue	61.16 ± 23.58	65.21 ± 21.76	58.33 ± 24.70	0.287
Emotional wellbeing	67.76 ± 15.46	70.26 ± 15.10	66.03 ± 15.70	0.318
Social functioning	73.97 ± 21.37	79.34 ± 20.50	70.22 ± 21.46	0.117
Pain	72.63 ± 19.50	82.50 ± 18.01	65.75 ± 17.67	0.001 *
General health	51.07 ± 24.13	60.86 ± 21.51	44.24 ± 23.78	0.010 *

HRQOL = Health-Related Quality of Life; CLDQ = Chronic Liver Disease Questionnaire; SF-36 = Short Form-36; * *p* < 0.05, ANOVA.

**Table 7:** Assessment of HRQOL among patients with cirrhosis.

HRQOL Parameters	Compensated (*n* = 11)	Decompensated (*n* = 22)	*p*-Value
CLDQ (mean ± SD)			
Total score	4.19 ± 0.89	2.63 ± 0.69	<0.001 *
Abdominal symptoms	3.87 ± 1.03	2.22 ± 0.63	<0.001 *
Fatigue	4.07 ± 1.09	2.60 ± 0.73	<0.001 *
Systemic symptoms	3.91 ± 0.92	2.61 ± 0.79	<0.001 *
Activity	4.35 ± 1.09	2.58 ± 1.01	<0.001 *
Emotional function	4.89 ± 1.14	3.22 ± 0.87	<0.001 *
Worry	4.07 ± 1.20	2.57 ± 1.01	0.001 *
**SF-36 (%, mean ± SD)**			
Physical functioning	84.09 ± 7.68	57.72 ± 16.88	<0.001 *
Role limitations due to physical health problems	84.09 ± 12.61	46.81 ± 21.63	<0.001 *
Role limitations due to emotional problems	75.78 ± 15.55	49.99 ± 19.94	0.001 *
Energy fatigue	76.36 ± 14.33	49.31 ± 24.01	0.002 *
Emotional wellbeing	78.81 ± 12.71	59.63 ± 13.05	<0.001 *
Social functioning	90.22 ± 10.33	60.22 ± 18.35	<0.001 *
Pain	83.40 ± 11.02	56.93 ± 13.15	<0.001 *
General health	61.36 ± 18.31	35.68 ± 21.72	0.001 *

HRQOL = Health-Related Quality of Life; CLDQ = Chronic Liver Disease Questionnaire; SF-36 = Short Form-36; * *p* < 0.05, ANOVA.

**Table 8:** Correlations between sleep assessment and HRQOL among enrolled patients.

HRQOL	PSQI	ESS	Sleep Efficacy	Number of Awakenings/Nights
**CLDQ (mean ± SD)**				
Total score	−0.671	−0.729	0.785	−0.769
Abdominal symptoms	−0.608	−0.671	0.724	−0.735
Fatigue	−0.670	−0.711	0.763	−0.768
Systemic symptoms	−0.644	−0.705	0.746	−0.741
Activity	−0.691	−0.751	0.819	−0.753
Emotional function	−0.571	−0.625	0.688	−0.674
Worry	−0.597	−0.650	0.689	−0.665
**SF-36 (mean %)**				
Physical functioning	−0.804	−0.809	0.711	−0.693
Role limitations due to physical health problems	−0.741	−0.825	0.634	−0.732
Role limitations due to emotional problems	−0.653	−0.669	0.566	−0.565
Energy fatigue	−0.667	−0.632	0.488	−0.502
Emotional wellbeing	−0.648	−0.595	0.517	−0.519
Social functioning	−0.735	−0.732	0.637	−0.63
Pain	−0.735	−0.752	0.716	−0.612
General health	−0.690	−0.682	0.585	−0.635

Values are correlation coefficients (Spearman’s r); HRQOL = Health-Related Quality of Life; CLDQ = chronic liver disease questionnaire; SF-36 = Short Form-36; PSQI = Pittsburgh Sleep Quality Index; ESS = Epworth Sleepiness Scale.

## Data Availability

Study data are available from the first author (O.-M.P.) upon request.
